# AI-based multimodal prediction of lymph node metastasis and capsular invasion in cT1N0M0 papillary thyroid carcinoma

**DOI:** 10.3389/fendo.2025.1580885

**Published:** 2025-05-27

**Authors:** Xiaowei Peng, Peng Wu, Wu Li, Tao Ou-Yang, Shi Chu Tang, Shiwei Zhou, Hui Li, Xiaohua Song, Yulong Tang

**Affiliations:** ^1^ Department of Thyroid Surgery, Hunan Cancer Hospital & The Affiliated Cancer Hospital of Xiangya School of Medicine, Central South University, Changsha, China; ^2^ Department of Medical Ultrasound, Hunan Cancer Hospital & The Affiliated Cancer Hospital of Xiangya School of Medicine, Central South University, Changsha, China

**Keywords:** papillary thyroid cancer, artificial intelligence, ultrasound radiomics, prediction model, risk stratification

## Abstract

**Background:**

Accurate preoperative evaluation of cT1N0M0 papillary thyroid carcinoma (PTC) is essential for guiding appropriate treatment strategies. Although ultrasound is widely used for clinical staging, it has limitations in detecting lymph node metastasis (LNM) and capsular invasion (CI), which may lead to misclassification of high-risk patients. Such undetected risks pose safety concerns for those undergoing radiofrequency ablation. This study aimed to develop an artificial intelligence (AI)-assisted predictive model that integrates ultrasound radiomics and deep learning features to improve the identification of LNM and CI, thereby enhancing risk stratification and optimizing treatment strategies for cT1N0M0 PTC patients.

**Methods:**

A total of 203 PTC patients were divided into high-risk (CI or LNM) and low-risk groups, with 142 assigned to the training set and 61 to the internal test set. Regions of interest delineation was performed using ITK-Snap. Radiomic features were extracted with PyRadiomics, and embedding features were obtained through the Vision Transformer (ViT) model. Risk-related features were selected using least absolute shrinkage and selection operator (LASSO), variance thresholding, and recursive feature elimination (RFE). Single-modal and multimodal models were developed using feature-level and decision-level fusion. Feature importance was assessed using Shapley Additive exPlanations (SHAP). Model performance was evaluated using recall, accuracy, and area under curve (AUC).

**Results:**

Among 1,001 radiomics features, 47 were selected via LASSO and RFE, and 15 relevant features from 768 ViT features. In the internal test set, NeuralNet models based on radiomics and 2D deep learning achieved AUCs of 0.756 and 0.708, respectively, and 0.829 and 0.840 in the training set. The multimodal RandomForest model outperformed single-modality models, with an AUC of 0.763 in the test set and 0.992 in the training set. Decision-level fusion models, such as DLRad_LF_Avg and DLRad_LF_Max, improved the external test set AUC to 0.843. SHAP analysis identified key features linked to tumor heterogeneity.

**Conclusion:**

The multimodal AI model effectively predicts high-risk cT1N0M0 PTC, outperforming single-modality models and aiding clinical decision-making.

## Introduction

In 2022, over 821,000 new thyroid cancer cases were reported globally, ranking it the 7th most common cancer, with women affected at nearly three times the rate of men. Despite its high incidence, the mortality rate was low, with around 44,000 deaths (1). This is mainly due to the widespread use of imaging and biopsy, which have increased the incidence of papillary thyroid cancer (PTC) (2). The management of low-risk PTC has been debated, especially regarding overtreatment. Recently, some researchers have suggested radiofrequency ablation (RFA) as a viable treatment option (3). RFA is a minimally invasive treatment technique that uses heat generated by high-frequency electrical currents to target and destroy diseased tissue. Although studies have evaluated the short-term clinical safety and efficacy of RFA in treating solitary T1N0M0 PTC, the indications for RFA in low-risk PTC patients have not yet been standardized (4–7).

Currently, research on the application of RFA in PTC primarily focuses on patients with T1N0M0 staging (5, 8). Clinical and pathological staging are critical for cancer management. Clinical staging, based on preoperative evaluations like ultrasound (US) and biopsy, guides treatment decisions but is often limited by diagnostic methods or subjective interpretation, especially for lymph node assessment (9). Pathological staging, derived from histological analysis of postoperative specimens, provides more accurate evaluations of capsular invasion (CI) and lymph node metastasis(LNM)but requires surgery. Accurate preoperative staging is essential for tailoring treatment plans, avoiding unnecessary overtreatment in low-risk PTC patients, and identifying those at risk for CI or LNM to ensure timely and appropriate intervention.

Recent advancements in artificial intelligence (AI) are transforming the medical field, especially in disease diagnosis and prediction. Leveraging deep learning (DL) and machine learning, AI extracts complex features from medical images, enhancing diagnostic accuracy and consistency. In thyroid cancer research, AI is widely used in US analysis for lesion segmentation, distinguishing benign from malignant lesions, and risk stratification, providing new approaches to personalized treatment (10–13).

This study aims to develop a predictive model that integrates clinical and US features with AI-extracted imaging data. The model will identify risk indicators for LNM or CI in cT1N0M0 PTC patients. It will assist clinicians in making informed decisions regarding treatment plans, identifying candidates for ablation or surgery, and optimizing RFA indications and management strategies.

## Materials and methods

### Patients and baseline information

The study included PTC patients (cT1N0M0) who underwent surgery at Hunan Cancer Hospital from January 2019 to July 2024. The retrospective study was approved by the Ethics Committee of Hunan Cancer Hospital. Patients were randomly split into training and internal test sets at a 7:3 ratio. To mitigate class imbalance in the dataset, the Synthetic Minority Oversampling Technique (SMOTE) was employed to achieve a balanced distribution between low-risk and high-risk patients. Clinical stage cT1N0M0 was defined by preoperative computed tomography or US showing no significant CI or cervical LNM, with tumor size ≤2 cm and no evidence of distant metastasis. US images were reviewed by a senior radiologist (S.-C.T.) with over 30 years of experience.

**Figure 1:**
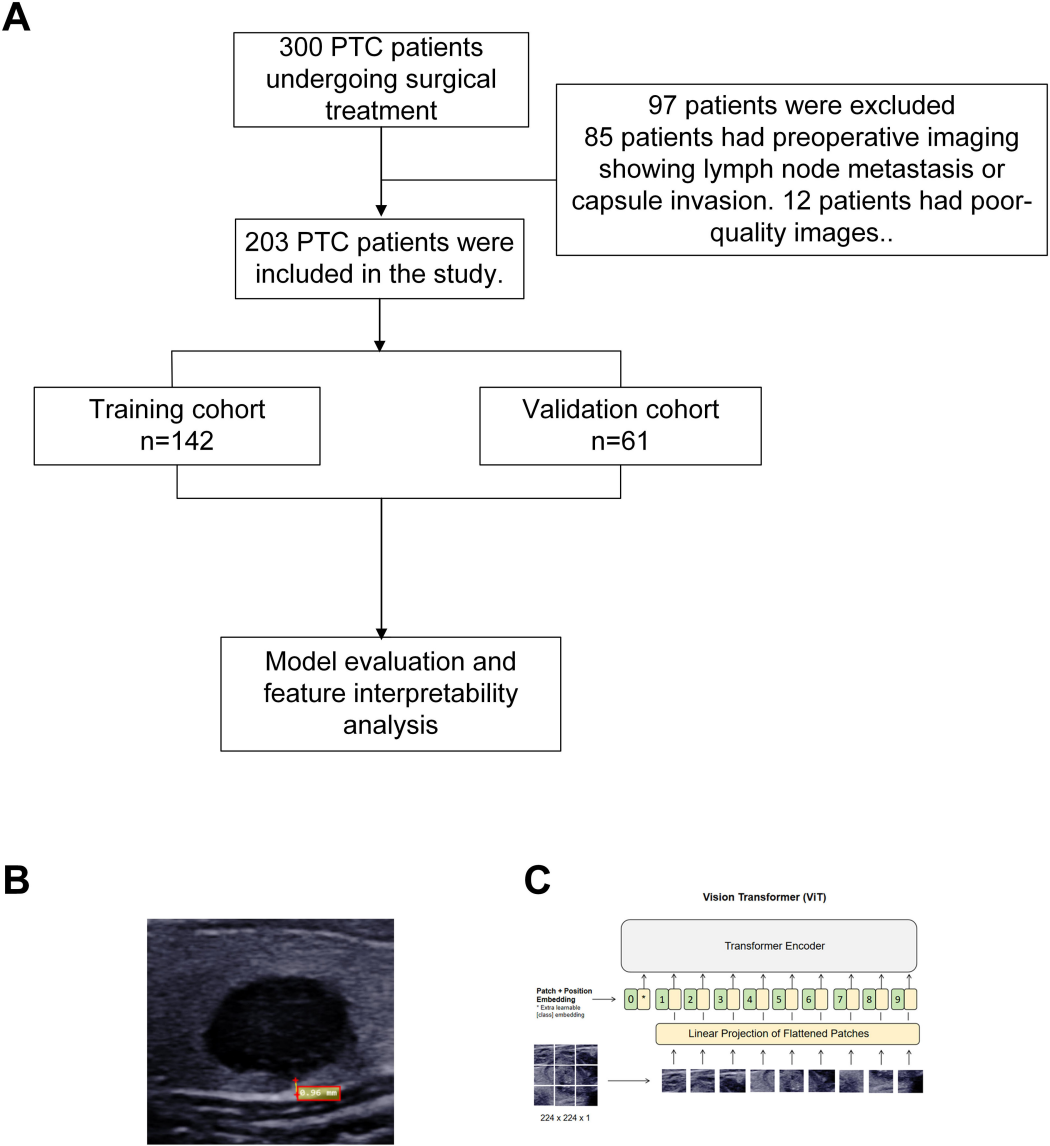
Study workflow and imaging analysis: **(A)** Study population flowchart, **(B)** Measurement of the shortest distance between the nodule and capsule using MicroDicom Viewer software on DICOM ultrasound images, **(C)** Feature extraction from entire image using deep learning approach. DICOM, digital imaging and communications in medicine; PTC, Papillary Thyroid Carcinoma.

Inclusion criteria: (i) complete clinical data; (ii) high-quality US images recognizable by AI. Exclusion criteria: (i) incomplete clinical or pathological data; (ii) poor-quality or AI-incompatible US images; (iii) preoperative evidence of LNM on CT or US; (iv) multifocal cancers. The study flowchart is shown in **Figure 1A**.

Postoperative pathology reports confirming CI or LNM were identified as risk factors. Baseline clinical and US data, including tumor size, age, gender, Hashimoto’s thyroiditis, benign nodules, nodule location, microcalcification, aspect ratio, nodule echo, tumor-to-capsule distance, and Color Doppler flow imaging (Adler grading system), were retrieved from medical records. The shortest tumor-to-capsule distance was measured using MicroDicom viewer (https://www.microdicom.com/) on US images (**Figure 1B**).

### US image acquisition

B-mode US and color Doppler flow images were obtained using a Super Sonic Aixplorer system (Super Sonic Imagine, Aix-en-Provence, France) with a 5–14 MHz linear transducer. The patient was placed in a supine position on the examination table with the neck slightly extended. The head was tilted backward to expose the thyroid gland and allow optimal imaging of both the right and left lobes. US scanning typically began at the lower part of the neck and continued upward to the thyroid isthmus. Both transverse and sagittal planes were imaged. All images were acquired by a senior radiologist (S.-C.T.) in the Hunan Cancer Hospital. One US image with the largest diameter of each tumor was used for analysis.

### ROI delineation and intraclass correlation coefficient analysis

An experienced (T.O-Y.) independently delineated the tumor regions using the ITK-Snap 3.8 software (http://www.itksnap.org) in a blinded manner to define the regions of interest (ROI). The delineated ROI primarily focused on the demarcation of the primary tumor area. To systematically assess the reliability and consistency of our ROI delineation, 50 patient cases were randomly selected for a second round of ROI delineation two weeks later. A thyroid surgeon (Y.-L.T.) trained in thyroid US independently annotated the tumor regions for the same 50 patients during this process. Inter- and intra-rater reliability were subsequently evaluated using ICC analysis.

### US image feature extraction based on PyRadiomics

Quantitative imaging features were extracted from US images using PyRadiomics (v3.0.1, https://github.com/Radiomics/pyradiomics) in Python (v3.10.14). Features were derived from original and mathematically transformed images, including Laplacian of Gaussian filtering, wavelet, exponential, square, square root, and logarithmic transformations. Extracted features included first-order statistics, Gray Level Co-occurrence Matrix, Gray Level Size Zone Matrix, Neighboring Gray Tone Difference Matrix, Gray Level Dependence Matrix, and others. Statistical features (e.g., mean, variance) described global properties, while texture features (e.g., Gray Level Co-occurrence Matrix) captured local variations. All analyses used PyRadiomics, with detailed feature definitions available at https://pyradiomics.readthedocs.io/en/latest/radiomics.html.

### US image embedding feature extraction based on vision transformer

US image embedding features were extracted using a pre-trained ViT-B/16 model (14). The model includes a convolutional projection layer to extract initial features, an encoder with self-attention mechanisms to capture long-range dependencies and local patterns, and a linear classifier head to map features to specific labels. The pre-trained weights were obtained from the ImageNet-1k dataset. To adapt the model to the current prediction task, we modified the classification head and incorporated a dropout layer with a dropout rate of 0.5. During fine-tuning, we used the Adam optimizer with an initial learning rate of 1e-3 and applied a ReduceLROnPlateau scheduler to dynamically adjust the learning rate (with a decay factor of 0.1 triggered after five consecutive epochs without improvement in validation loss). The batch size was set to 32, and the weight decay coefficient was 0.01. In this study, the convolutional layer extracted features, and the encoder output, excluding the classification head, was used to generate image embeddings with rich semantic and contextual information for subsequent analysis and diagnosis (**Figure 1C**).

### Feature selection

Feature selection was performed on features extracted from the ViT pre-trained model and PyRadiomics. First, the variance threshold method was applied to remove features with a variance below 0.05 to reduce noise. The remaining features were then standardized using the z-score method (mean = 0, standard deviation = 1). Subsequently, least absolute shrinkage and selection operator (LASSO) regression and Recursive Feature Elimination (RFE) were employed to identify features associated with risk factors. Finally, the union of features selected by LASSO and RFE, with an ICC greater than 0.75, was used for subsequent model construction. LASSO was implemented using the glmnet package (v4.1_8), and RFE was performed using the caret package (v6.0_94). Feature correlations were visualized using the pheatmap package (v1.0.12).

### Construction and evaluation of models

In this study, two multimodal fusion strategies were employed to construct predictive models (1): Feature-level fusion (early fusion, DLRad_EF): Radiomic features extracted using PyRadiomics were concatenated with 2D DL features derived from a fine-tuned ViT model to form a unified feature vector, which was then input into classifiers for retraining. This approach enables integration of multimodal information at the feature level, aiming to exploit complementary information between the two modalities and enhance the model’s ability to recognize complex patterns (2). Decision-level fusion (late fusion, DLRad_LF): The output probabilities of the Radiomics and 2D-DL models were combined using ensemble strategies such as maximum and averaging operations, leveraging the strengths of each single-modality model to improve predictive accuracy and stability. For both single-modality and DLRad_EF models, we employed a variety of advanced machine learning algorithms, including NeuralNet, XGBoost, LightGBM, CatBoost, ExtraTrees, RandomForest, and KNeighbors. This study employed a comprehensive evaluation approach based on multiple metrics. The performance of the Radiomics, 2D-DL, DLRad_EF, and DLRad_LF models was thoroughly compared using several key metrics, including accuracy, recall, precision, F1-score, model complexity.

### Feature interpretability analysis

Shapley Additive exPlanations (SHAP) was used to explain feature importance. SHAP is a method for interpreting machine learning models based on Shapley value theory from game theory. It decomposes the contribution of each feature to the prediction outcome, providing a relative importance ranking for each feature. SHAP can generate importance rankings for individual samples, individual features, or feature combinations, which is useful for understanding the overall behavior of the model and the influence of specific features on a given prediction.

### Statistical analysis

Analyses were conducted using R software version 4.4.1 (https://www.r-project.org/) and Python software version 3.10.14 (https://www.python.org/). The Mann–Whitney U test was employed to compare characteristics among different groups for continuous variables (not normally distributed), while the independent samples t-test was utilized for continuous variables that followed a normal distribution. The chi-square test was applied to assess differences in categorical variables. All levels of statistical significance are bilateral, with a *P* value less than 0.05.

## Results

### Patient characteristics

A total of 203 PTC patients who underwent surgical treatment between January 2019 to July 2024 at Hunan Cancer Hospital were included in the study. The training cohort consisted of 142 patients (median age: 41.50 years [31.00, 49.25]; age range: 12–65 years), including 62 patients with risk factors (22 with CI and 51 with LNM) and 80 patients without risk factors. The validation cohort included 61 patients (median age: 42.00 years [33.00, 50.50]; age range: 21–58 years), comprising 23 patients with risk factors (8 with CI and 17 with LNM) and 38 patients without risk factors. There was no significant difference in composition ratio of patients between the two cohorts. The baseline characteristics of patients in the training and validation cohorts are shown in **Table 1**. Univariate analysis was performed using the clinical and US features of patients in the training cohort. The results showed that tumor diameter and tumor location (whether located in the upper pole) exhibited significant differences between the invasive group and non-invasive groups (**Table 2**).

**Table 1:** Clinical and ultrasound information of patients in the training and validation cohorts.

Characteristic	Training Cohort (n=142)	Validation Cohort (n=61)	*P value*
Age, median ± interquartile range, years	[41.50 (31.00, 49.25)]	[42.00(33.00, 50.50)]	0.630‖
Capsular invasion or lymph node metastasis			0.443§
Positive	62 (43.66)	23 (37.70)	
Negative	80 (56.34)	38 (62.30)	
Gender			0.393§
Male	42 (29.58)	14 (22.95)	
Female	100 (70.42)	47 (77.05)	
Microcalcification			0.539§
Positive	62 (43.66)	30 (49.18)	
Negative	80 (56.34)	31 (50.82)	
Diameter, median ± interquartile range, cm	[0.80 (0.60, 1.10)]	[0.80(0.70, 1.10)]	0.976‖
Aspect ratio			0.647§
>1	76 (53.52)	35 (57.38)	
≤1	66 (46.48)	26 (42.62)	
Hashimoto’s thyroiditis			0.484§
Positive	34 (23.94)	18 (29.51)	
Negative	108 (76.06)	43 (70.49)	
With benign lesions			0.424§
Positive	47 (33.10)	24 (39.34)	
Negative	95 (66.90)	37 (60.66)	
Nodule location			0.802§
Upper	14 (9.86)	7 (11.48)	
Other location	128 (90.14)	54 (88.52)	
CDFI (Adler grading system)			0.558§
Grade 0 (avascular)	19 (13.38)	5 (8.20)	
Grade I (minimal)	75 (52.81)	37 (60.66)	
Grade II (moderate)	30 (21.13)	10 (16.39)	
Grade III (marked)	18 (12.68)	9 (14.75)	
Nodule echo			0.714§
Very hypoechoic	10 (7.04)	5 (8.20)	
Hypoechoic	128 (90.14)	53 (86.88)	
Hyperechoic or mixed echogenicity	4 (2.82)	3 (4.92)	
Distance from tumor to thyroid capsule, median ± interquartile range, mm	[0.91 (0.51, 1.48)]	[1.03(0.54, 1.40)]	0.677‖

Data expressed as n (%), unless otherwise stated.

CDFI, color Doppler flow imaging.

§By the Chi-square test.

‖By the Mann–Whitney U test.

**Table 2:** Clinical characteristics of patients in the training cohorts.

Characteristic	Non-invasive Group (n=80)	Invasive Group (n=62)	*P value*
Age, mean ± SD, years	41.50 ± 10.30	40.47 ± 10.79	0.563¶
Gender			0.197§
Male	20 (25.00)	22 (35.48)	
Female	60 (75.00)	40 (64.52)	
Microcalcification			0.197§
Positive	32 (40.00)	30 (48.39)	
Negative	48 (60.00)	32 (51.61)	
Diameter, median ± interquartile range, cm	[0.80(0.60, 1.00)]	[0.90(0.70, 1.20)]	0.011‖
Aspect ratio			0.127§
>1	38 (5.74)	38 (13.33)	
≤1	42 (94.26)	24 (86.67)	
Hashimoto’s thyroiditis			0.074§
Positive	24 (33.61)	10 (35.00)	
Negative	56 (66.39)	52 (65.00)	
With benign lesions			0.596§
Positive	24 (30.00)	10 (16.13)	
Negative	56 (70.00)	52 (83.87)	
Nodule location			0.001§
Upper	2	15	
Other location	78	47	
CDFI (Adler grading system)			0.538§
Grade 0 (avascular)	13 (16.25)	6 (9.68)	
Grade I (minimal)	42 (52.50)	33 (53.23)	
Grade II (moderate)	17 (21.25)	13 (20.96)	
Grade III (marked)	8 (10.00)	10 (16.13)	
Nodule echo			0.719§
Very hypoechoic	6 (7.50)	4 (6.45)	
Hypoechoic	71 (88.75)	57 (91.94)	
Hyperechoic or mixed echogenicity	3 (3.75)	1 (1.61)	
Distance from tumor to thyroid capsule, median ± interquartile range, mm	[0.91(0.50, 1.46)]	[0.93(0.52, 1.50)]	0.858‖

Data expressed as n (%), unless otherwise stated.

CDFI, color Doppler flow imaging.

¶By the Independent samples t-test.

§By the Chi-square test.

‖By the Mann–Whitney U test.

### Feature selection and model construction

**Figure 2:**
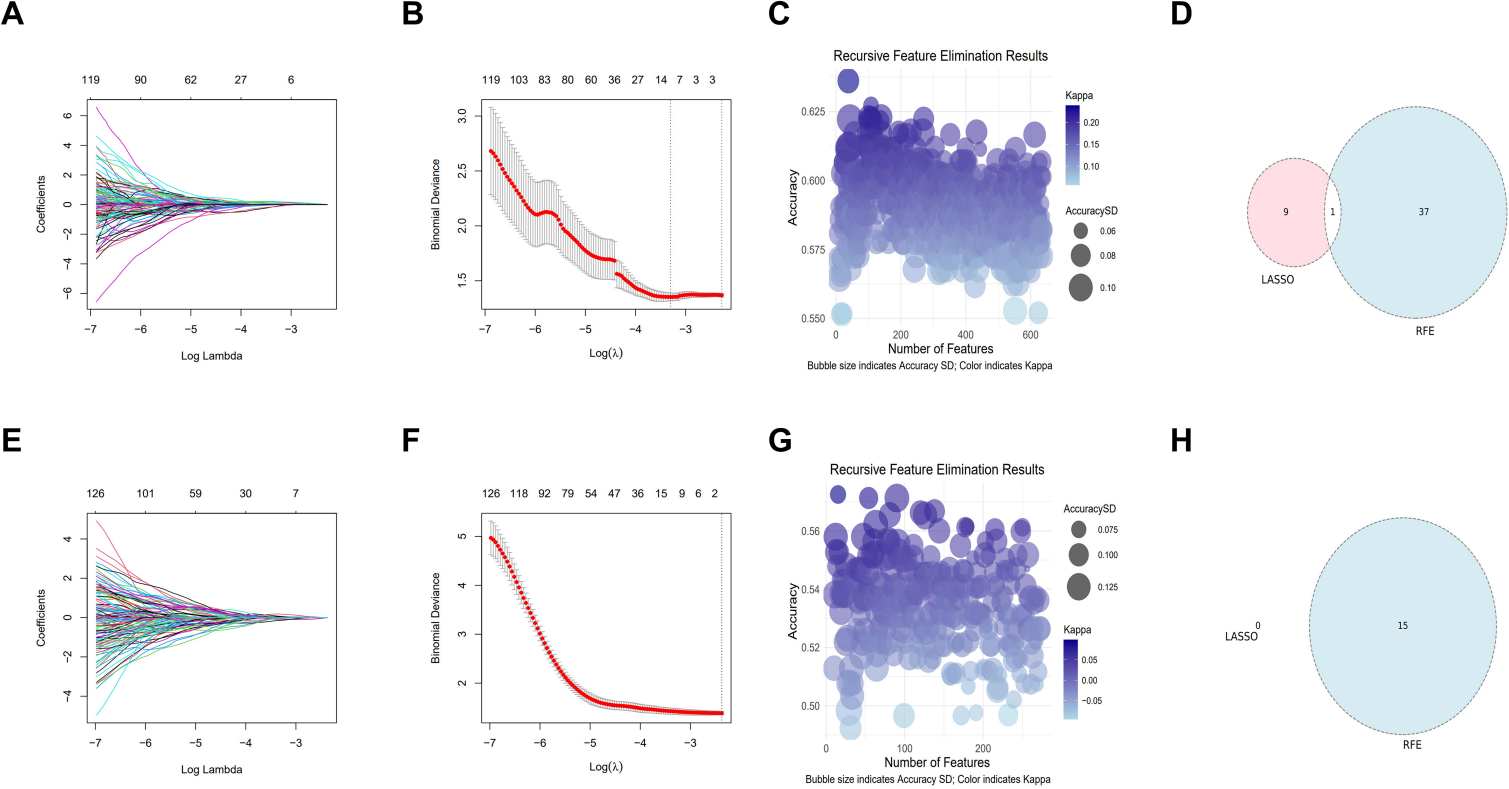
Feature selection process for radiomics and vision transformer (ViT) features: **(A**, **B)** LASSO regression for radiomics features, **(C)** RFE accuracy versus number of selected radiomics features, **(D)** Union set of LASSO/RFE-selected radiomics features, **(E**, **F)** ViT-derived feature LASSO analysis (no features met criteria), **(G**, **H)** ViT feature RFE curve with peak accuracy at 15 features. LASSO, Least Absolute Shrinkage and Selection Operator; RFE, Recursive Feature Elimination; SD, standard deviation.

Total of 1001 radiomics features were extracted, with 980 stable features retained (ICC > 0.75). Using LASSO with lambda.min, 10 features were selected, while RFE identified 38 features most associated with risk factors. LASSO and RFE selection processes are shown in **Figures 2A–D**, and their union was used for further analysis. For the 768 imaging features extracted via the ViT model, similar processing was applied. **Figures 2E–H** illustrate the feature selection, where LASSO identified no features, and 15 RFE-selected features were used for model construction. Final features are detailed in **Supplementary Table 1**.

**Figure 3:**
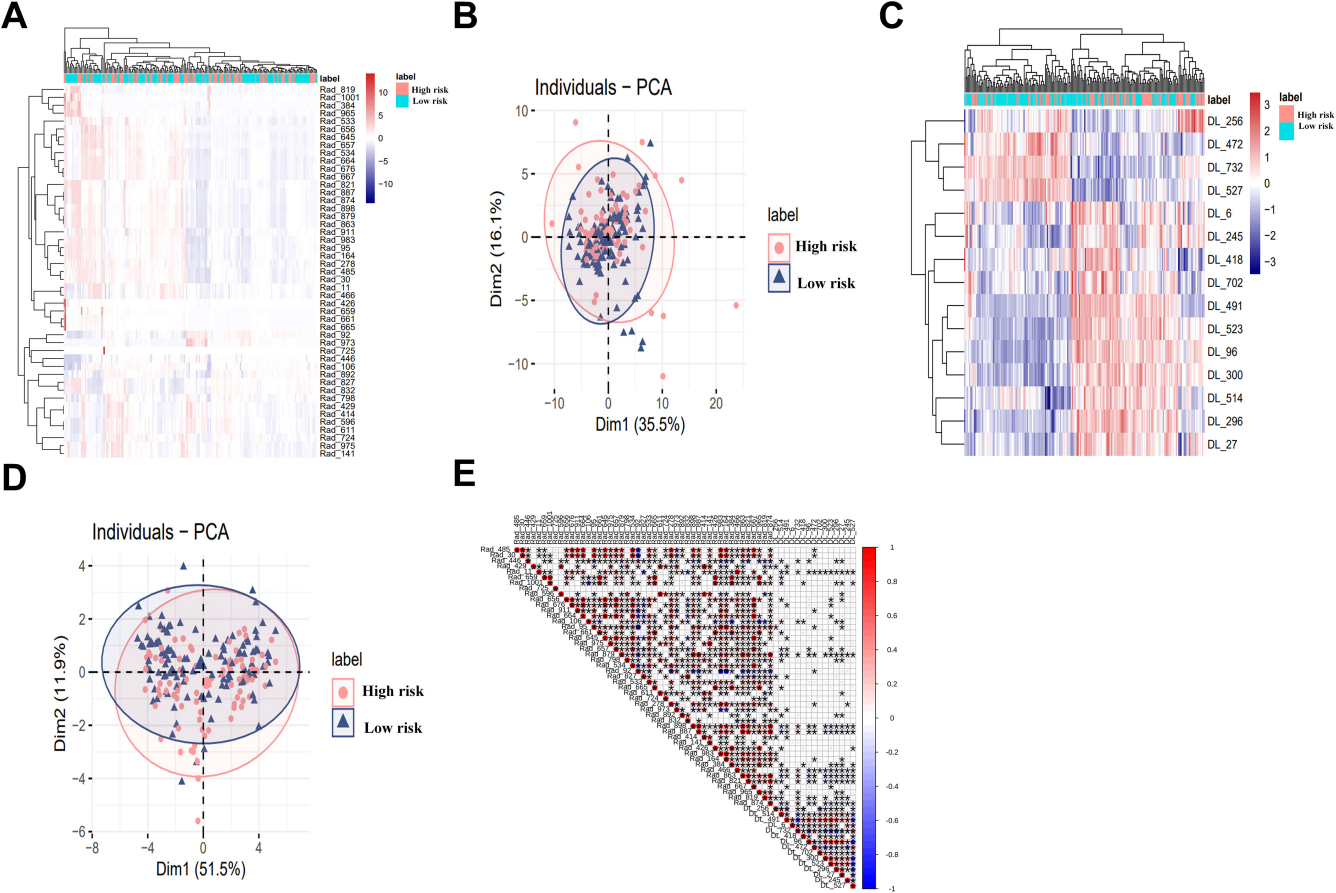
Radiomics and DL features for high-risk patient identification and correlation analysis: **(A-D)** Heatmap and PCA were applied for feature dimensionality reduction and visualization. Figures **(A, B)** show the relationship between radiomics features and high-risk status, while Figures **(C, D)** display the relationship between 2D DL features and high-risk status. **(E)** Spearman correlation analysis revealed significant redundancy between radiomics and 2D deep learning features. The size of the circles represents the absolute value of the correlation coefficient, while the color of the circles indicates the direction of the correlation: red for positive correlation and blue for negative correlation, *P<0.05. DL, deep learning, PCA, Principal Component Analysis.

Principal component analysis and heatmaps were used for dimensionality reduction and visualization of selected features. **Figures 3A–D** depict associations between radiomics and 2D DL features with risk factors. Spearman correlation analysis (**Figure 3E**) revealed redundancy within radiomics and DL features, but limited correlation between the two, indicating complementary information. In multivariate analysis integrating clinical and ultrasound features with radiomics, tumor diameter and location (*P* > 0.05) were excluded from the final predictive model.

### Comparison of model performance

**Figure 4:**
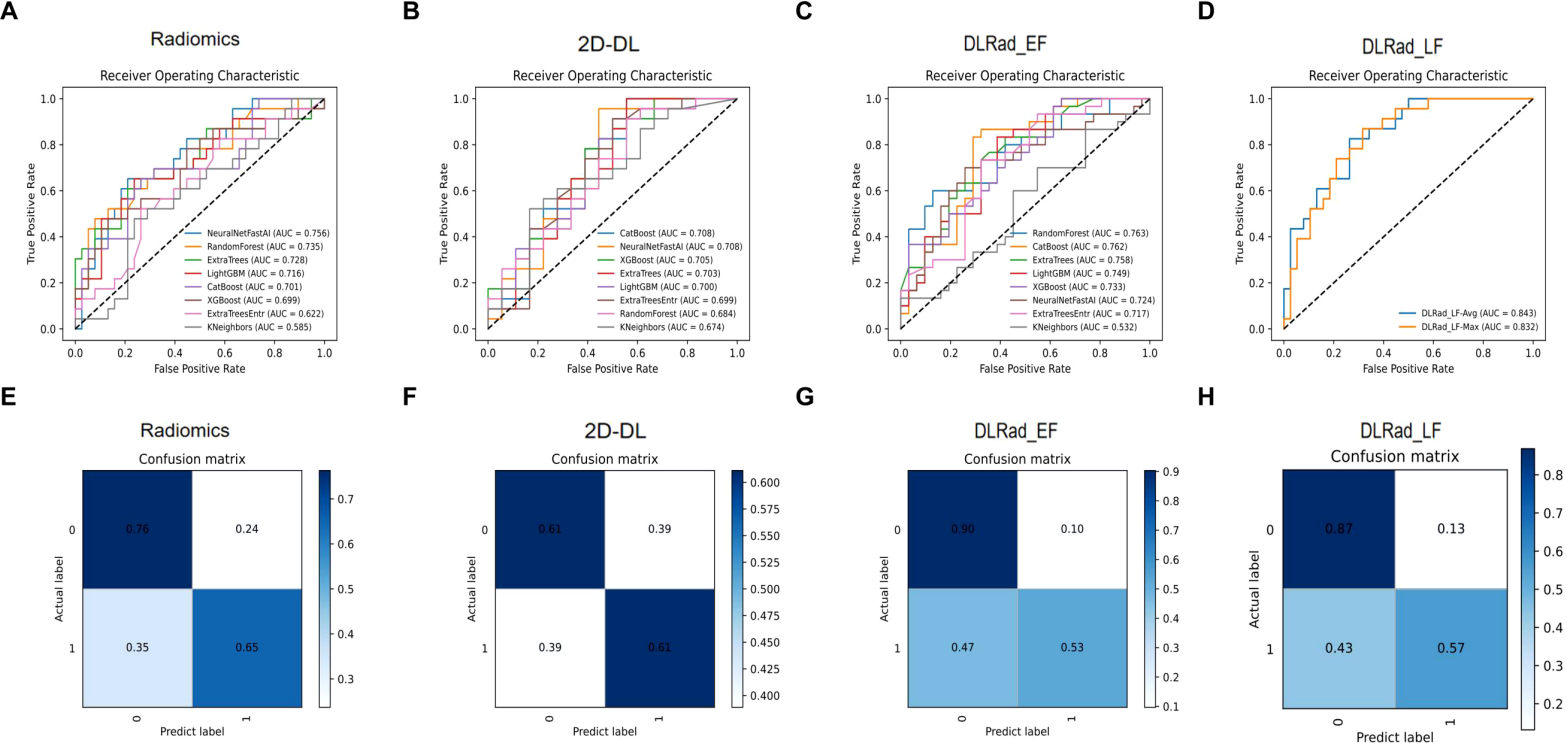
Comparison of model performance: **(A, B)** The NeuralNet method achieved the best performance among models trained with radiomics features and 2D deep learning features on the test set. **(C)** RandomForest exhibited superior performance in the multimodal model combining radiomics and 2D deep learning features. **(D)** The average ensemble of the two single-modality models improved the AUC on the external test set to 0.843. **(E-H)** Confusion matrices display the prediction accuracy of the models on the test set. AUC, area under the curve; DL, deep learning; DLRad_EF, deep learning radiomics early fusion; DLRad_LF, deep learning radiomics late fusion.

High-risk thyroid patients were predicted using various machine learning algorithms, evaluated by receiver operating characteristic curves, confusion matrices, and metrics such as recall, precision, accuracy, and F1 score. In the internal test set, NeuralNet achieved the best performance among models trained on radiomics and 2D DL features, with area under the curve (AUC) values of 0.756 and 0.708, respectively, and was selected for further analysis (**Figures 4A, B**). For multimodal models combining radiomics and 2D DL features, RandomForest outperformed others, resulting in the final DLRad_EF model with an AUC of 0.763, slightly better than single-modality models due to complementary feature information, despite some redundancy (**Figure 4C**).

DLRad_LF_Avg and DLRad_LF_Max, decision-level fusion methods based on averaging and maximum output values, improved the external test set AUC to 0.843 through ensemble predictions of the single-modality models (**Figure 4D**). The confusion matrix of the best model showed a 76% correct classification rate for low-risk patients and a 65% success rate for identifying high-risk patients (**Figures 4E–H**).

### Comparison of performance based on different data

**Figure 5:**
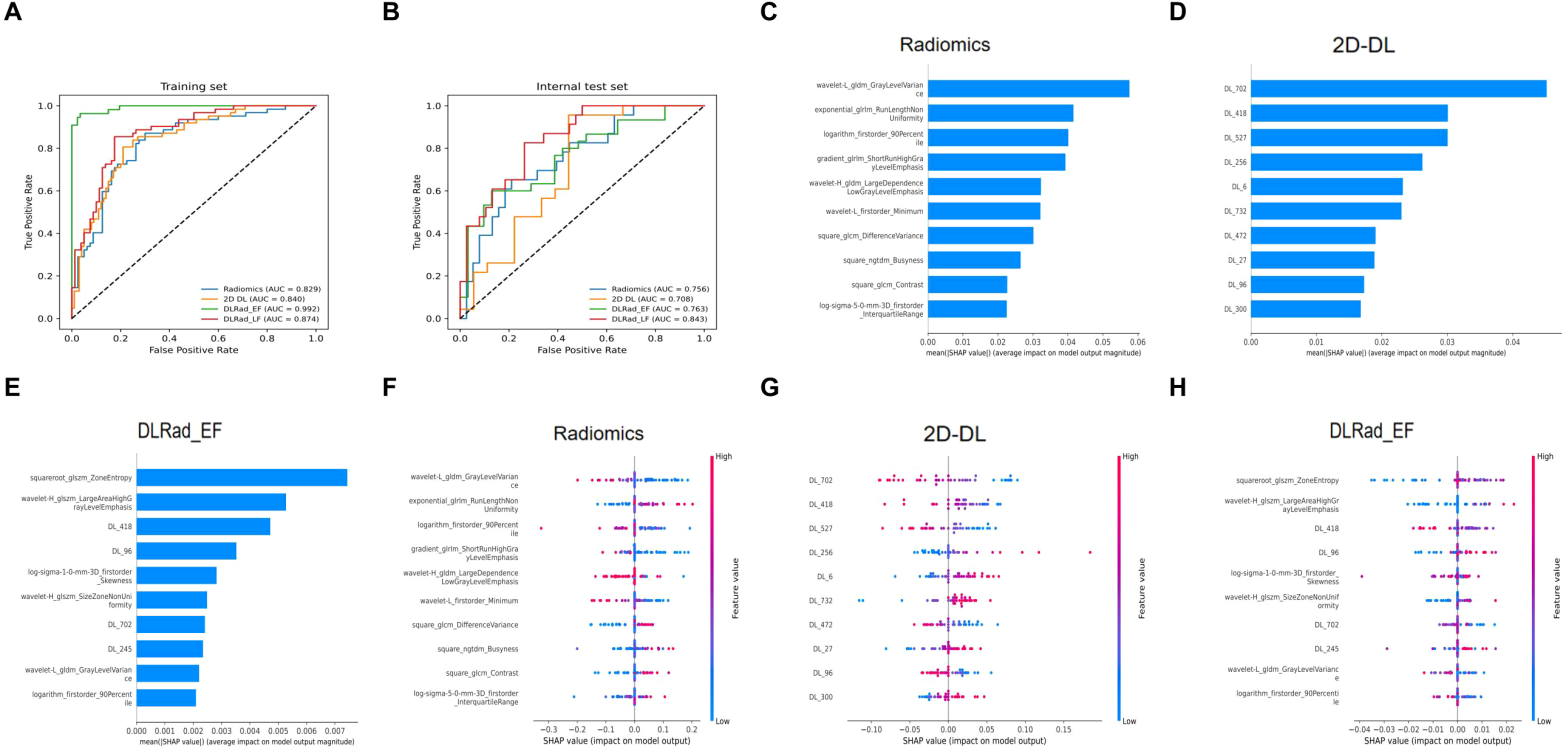
Performance comparison across different datasets and SHAP-based interpretability analysis: **(A)** Model performance on the training set. **(B)** Model performance on the test set. SHAP analysis was applied to the radiomics model **(C**–**F)**, 2D DL model **(D**–**G)**, and DLRad_EF model **(E**–**H)** to visualize the top 10 most contributing features. AUC, area under the curve; DL, deep learning; DLRad_EF, deep learning radiomics early fusion; DLRad_LF, deep learning radiomics late fusion; SHAP, SHapley Additive exPlanations.

We compared the AUC performance of single-modality models using radiomics and DL features, as well as multimodal models with DLRad_EF and DLRad_LF. The results (**Figures 5A, B**) showed that the radiomics model had an AUC of 0.829 in the training set and 0.756 in the testing set, while the 2D DL model achieved AUCs of 0.840 and 0.708, respectively. DLRad_EF reached an AUC of 0.992 in the training set and 0.763 in the testing set, and DLRad_LF had AUCs of 0.874 and 0.843, respectively. A default threshold of 0.5 was used for model classification. Additional evaluation metrics are provided in **Table 3**. These results suggest that combining radiomics and 2D DL features allows effective prediction of high-risk cT1N0M0 PTC patients.

**Table 3:** Comparison of the performance of predictive models.

Model and metric	AUC, 95%CI	Accuracy	Precision	Recall	F1-score
Training set					
Radiomics	0.829 [0.761, 0.890]	0.754	0.696	0.774	0.731
2D DL	0.840 [0.779, 0.900]	0.772	0.712	0.677	0.694
DLRad_EF	0.992 [0.980, 0.999]	0.958	0.945	0.945	0.945
DLRad_LF	0.874 [0.819,0.925]	0.803	0.774	0.774	0.774
Internal test set					
Radiomics	0.756 [0.629, 0.870]	0.721	0.625	0.652	0.638
2D DL	0.708 [0.539, 0.858]	0.610	0.667	0.609	0.636
DLRad_EF	0.763 [0.632, 0.872]	0.721	0.842	0.533	0.653
DLRad_LF	0.843 [0.745, 0.934]	0.754	0.722	0.565	0.634

AUC, area under the curve; CI, confidence interval; DL, deep learning; DLRad_EF, DL Rad early fusion; DLRad_LF, DL Rad late fusion.

### Feature interpretability analysis

SHAP-based interpretability analysis was performed for the radiomics, 2D DL, and DLRad_EF models to rank feature importance. The top 10 contributing features were visualized in **Figures 5C–E**. For the radiomics model, certain first-order statistical and second-order texture features predicted CI or LNM. Key features like exponential_glrlm_RunLengthNonUniformity, square_glcm_DifferenceVariance, square_ngtdm_Busyness, and square_glcm_Contrast highlighted increased heterogeneity, rougher textures, and greater irregularity in high-risk tumors (**Figure 5F**).

**Figure 6:**
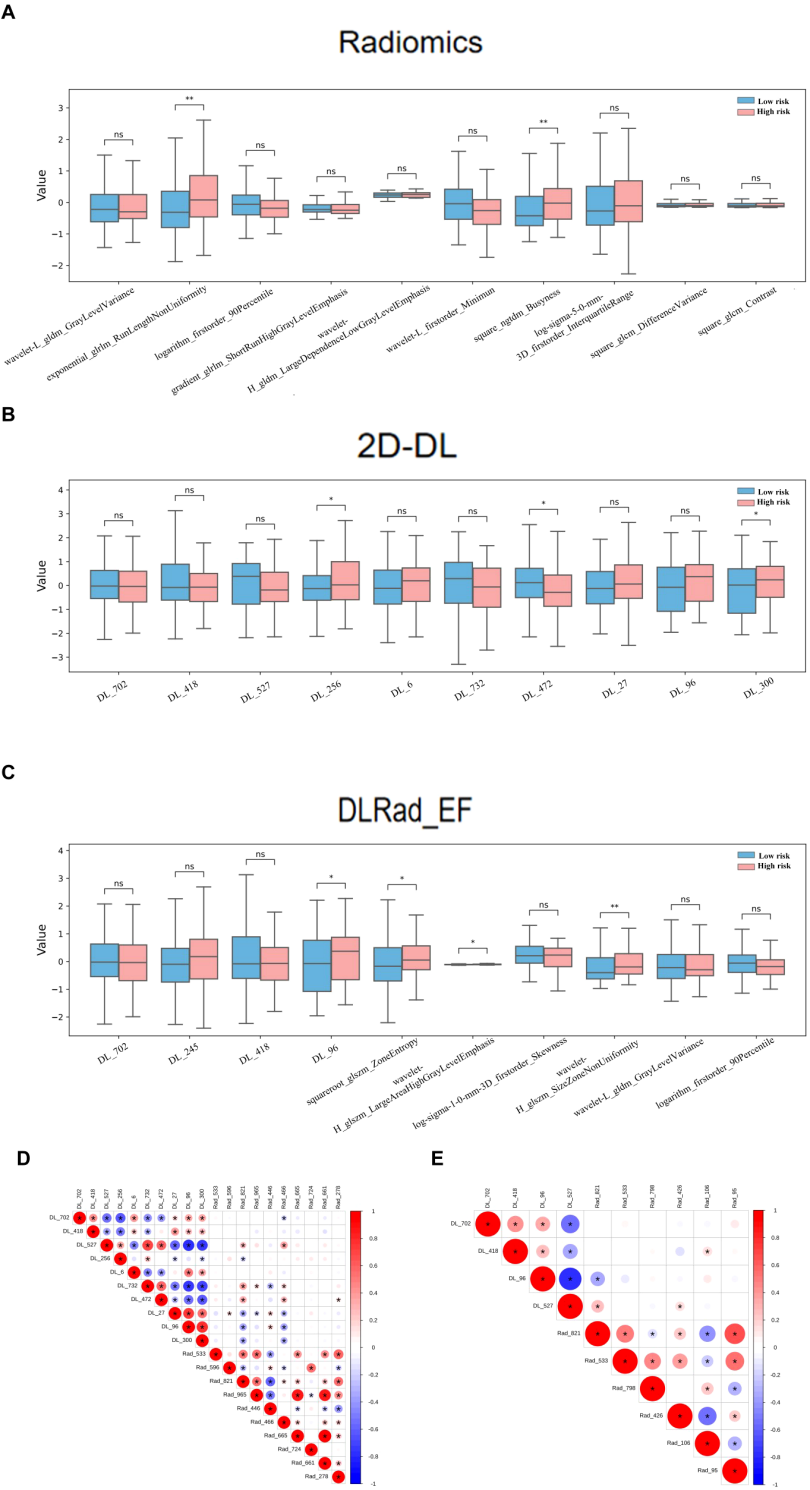
Comparison of the Top 10 SHAP features and their correlations across radiomics, 2D DL, and DLRad_EF Models in High-Risk and Low-Risk Groups: Boxplots of the top 10 SHAP features in the high-risk and low-risk groups for the radiomics model **(A)**, 2D DL model **(B)**, and DLRad_EF model **(C)**. Spearman correlation analysis revealed redundancy between the top 10 features of the radiomics and 2D DL models **(D)**, and the correlation among the top 10 features of the DLRad_EF model **(E)**. The size of the circles represents the absolute value of the correlation coefficient, while the color of the circles indicates the direction of the correlation: red for positive correlation and blue for negative correlation. *P < 0.05, **P < 0.01. DL, deep learning; DLRad_EF, deep learning radiomics early fusion; SHAP, SHapley Additive exPlanations.

In the DL-extracted features, lower values of DL_702, DL_418, DL_527, DL_472, and DL_96 and higher values of DL_256, DL_300, DL_6, DL_732, and DL_27 were linked to CI or LNM (**Figures 5G**). For the combined model, features indicating tumor heterogeneity, such as wavelet-H_glszm_SizeZoneNonUniformity, squareroot-glszm_ZoneEntropy, and DL_96, were strongly associated with high-risk tumors (**Figure 5H**). The box plots demonstrate the distributional differences of these features between low-risk and high-risk patients (**Figures 6A–C**).

Spearman analysis revealed redundancy within the top 10 features of the radiomics and 2D DL models (**Figure 6D**) and among the top features of the DLRad_EF model (**Figure 6E**). Features from different types were complementary, while those within the same type exhibited partial redundancy.

## Discussion

RFA has emerged as a promising minimally invasive treatment for low-risk PTC, particularly in cT1N0M0 patients. Several studies have shown that compared to traditional surgery, RFA achieves good outcomes in terms of local tumor control, reduced complications, and preservation of thyroid function (15–17). However, significant controversy remains regarding the safety and applicability of RFA in cT1N0M0 PTC patients, with key concerns centered around undetected LNM and CI, which could lead to disease progression or incomplete treatment (18, 19). Studies have shown that CI is a significant independent risk factor for LNM in PTC, with a stronger predictive value (20, 21). Subcapsular location (≤2 mm from capsule) was identified as an independent risk factor for local tumor progression after RFA (5). Besides, the routine use of prophylactic central neck dissection is advocated by many scholars, as it has been shown to significantly reduce local recurrence rates and reoperation rates, while facilitating accurate pathological staging to more precisely identify patients who may benefit from adjuvant therapy. This viewpoint is supported by studies indicating a high incidence of occult central LNM in patients with PTC (22, 23).

Current clinical guidelines recommend considering RFA for low-risk thyroid cancer patients who have undergone rigorous preoperative imaging evaluation (24). While these imaging techniques provide valuable insights, their sensitivity in detecting small LNM and CI remains suboptimal. Specifically, US is highly operator-dependent and has limited ability to visualize deep or retrotracheal lymph nodes, potentially leading to missed lymph node metastases. On the other hand, computed tomography offers superior depth penetration and may detect more extensive disease, but it still faces challenges in accurately characterizing smaller or less conspicuous metastatic lymph nodes, particularly in the central compartment (25, 26). These limitations underscore the need for more advanced diagnostic tools that can enhance both sensitivity and accuracy, thereby improving patient stratification and enabling more precise treatment decisions. Therefore, reliance on these imaging methods alone for preoperative staging may result in an underestimation of the true extent of disease, which could impact subsequent treatment planning and patient outcomes (27, 28).

The predictive value of clinical and ultrasonographic features in assessing LNM or CI in low-risk PTC remains without a unified standard. Existing evidence suggests that tumor size and its spatial relationship with key anatomical structures may serve as important predictors (29–31). Several studies, particularly those focused on RFA and active surveillance, have incorporated tumor size, capsular invasion, and other factors when determining appropriate management strategies for low-risk PTC patients (32, 33). In our study, tumor size and location in the upper pole were associated with LNM or CI in univariate analysis. However, in the multivariate analysis incorporating the AI model, the *P-*values were all greater than 0.05. This indicates that radiomic features substantially outperform traditional clinical and ultrasonographic characteristics in prediction.

Although several studies have utilized imaging or pathological features of thyroid lesions to predict cervical lymph node status or CI, few have focused on cT1N0M0 patients with PTC (34, 35). Most research has concentrated on papillary thyroid microcarcinoma (PTMC) patients, regardless of whether LNM or CI is suspected based on imaging or physical examination (36, 37). Given the ongoing debate regarding overtreatment versus conservative management in thyroid cancer, and the fact that current indications for active surveillance or RFA often pertain to this group of patients, our prediction model has demonstrated good diagnostic performance by leveraging radiomics features extracted from ultrasound images of tumors. It holds the potential to provide valuable insights for personalized treatment, precise risk stratification, and the formulation of evidence-based clinical guidelines for thyroid cancer.

The multimodal AI model in this study demonstrates distinct performance characteristics. For medical institutions favoring conservative treatment (such as active surveillance or radiofrequency ablation), the DLRad_EF model exhibits a higher positive predictive value (Precision=0.842), indicating its effectiveness in reducing unnecessary thyroidectomies. However, its relatively lower recall (Recall=0.533) suggests potential underdiagnosis risks. Conversely, in clinical settings prioritizing definitive treatment, the radiomics-based model (Recall=0.652) can reduce the likelihood of missing high-risk cases, though its lower positive predictive value (Precision=0.625) may lead to overtreatment. Notably, the decision-level fusion model DLRad_LF achieves a balanced performance in both AUC (0.843) and F1-score (0.634), offering a compromise for institutions needing to balance overtreatment and undertreatment risks.

The improved performance is supported by two technical considerations: (1) The integration of ViT-derived DL features, which provide global perspectives and inter-regional interaction patterns, with radiomics features that offer localized texture and morphological details, creates complementary feature representations; (2) A combined strategy of early feature fusion and late decision fusion was adopted to optimize model performance. Early feature fusion integrates DL and radiomics features at the feature level, enhancing the model’s representation and pattern recognition capabilities. Late decision fusion combines the output probabilities of single-modality models, leveraging their respective strengths to improve prediction accuracy and model robustness.

Our study has some limitations. Firstly, it is a retrospective study that collected data from only one hospital, which may introduce selection bias. Additionally, the relatively small sample size of 203 patients may raise concerns regarding the model’s generalizability and the risk of overfitting. To mitigate these issues, we employed data augmentation techniques, such as random rotation, flipping, and cropping, to increase the diversity of the training data and enhance the model’s ability to generalize to unseen data. Furthermore, we incorporated regularization methods, including Dropout and L2 regularization, to prevent overfitting. Dropout was applied to the fully connected layers, with a 50% probability of randomly dropping neurons during training, which forces the model to learn more robust and diverse features. L2 regularization, implemented through weight decay, helped constrain the model’s complexity by penalizing large weights, thereby promoting simpler, more generalizable models. While these techniques helped improve model robustness, we acknowledge that the limited sample size and the need for external validation remain challenges. We plan to expand the dataset and perform external validation using independent multicenter cohorts in future studies to enhance model diversity and further assess its clinical applicability.

This study developed and validated an AI-based multimodal predictive model integrating radiomics and 2D DL features to predict high-risk factors, including CI and LNM, in cT1N0M0 PTC patients. The model demonstrated robust predictive performance, with an ensemble approach yielding superior results compared to single-modality models. Our findings highlight the complementary value of combining radiomics and DL features, as these modalities capture distinct yet synergistic aspects of tumor heterogeneity and microenvironmental changes. It should be noted that in addition to its predictive accuracy and robustness, the model’s effective clinical integration further requires deployability and practical utility. In terms of computational resources, although model training relies on GPU acceleration, inference during deployment can be efficiently performed on standard CPU devices, with an average processing time of approximately 2–3 minutes per image. The model can be integrated into existing hospital information systems (e.g., Picture Archiving and Communication System), enabling automatic image import, preprocessing, feature extraction, and output of prediction results, thereby facilitating real-time clinical decision-making. Successful implementation also depends on interdisciplinary collaboration among AI engineers, clinicians, and information technology personnel, as well as structured training for physicians to enhance their understanding and appropriate use of AI tools. Moving forward, continuous incorporation of new data and clinical feedback will be essential for further optimizing model performance and improving its adaptability and scalability across diverse clinical settings.

## Data Availability

The raw data supporting the conclusions of this article will be made available by the authors, without undue reservation.
