# Supplementary material for: AI-based multimodal prediction of lymph node metastasis and capsular invasion in cT1N0M0 papillary thyroid carcinoma
Source: Front Endocrinol (Lausanne). 2025 May 27;16:1580885. doi: 10.3389/fendo.2025.1580885 (PMC12148903; doi:10.3389/fendo.2025.1580885)
Supplement: Supplementary file 3 [file Table1.docx]

Supplementary Table 1 The selected features for model construction.

| **Feature origin (N)** | **Feature names** |
| --- | --- |
| Radiomics (N = 47) | wavelet-L_glcm_JointEntropy |
|  | original_glcm_JointEntropy |
|  | wavelet-H_gldm_LargeDependenceLowGrayLevelEmphasis |
|  | wavelet-H_glszm_SizeZoneNonUniformity |
|  | original_firstorder_Minimum |
|  | square_glcm_ClusterShade |
|  | gradient_ngtdm_Strength |
|  | square_ngtdm_Coarseness |
|  | exponential_glrlm_RunLengthNonUniformity |
|  | square_glcm_Autocorrelation |
|  | square_glcm_SumEntropy |
|  | gradient_firstorder_10Percentile |
|  | square_glcm_DifferenceEntropy |
|  | log-sigma-1-0-mm-3D_firstorder_Skewness |
|  | log-sigma-1-0-mm-3D_firstorder_Entropy |
|  | square_glcm_Contrast |
|  | square_firstorder_MeanAbsoluteDeviation |
|  | gradient_glszm_SizeZoneNonUniformity |
|  | square_glcm_JointAverage |
|  | logarithm_glszm_HighGrayLevelZoneEmphasis |
|  | squareroot_glszm_ZoneEntropy |
|  | wavelet-L_gldm_HighGrayLevelEmphasis |
|  | log-sigma-1-0-mm-3D_firstorder_10Percentile |
|  | logarithm_firstorder_MeanAbsoluteDeviation |
|  | wavelet-L_gldm_GrayLevelVariance |
|  | square_glcm_DifferenceVariance |
|  | exponential_glszm_SizeZoneNonUniformity |
|  | square_ngtdm_Busyness |
|  | log-sigma-5-0-mm-3D_firstorder_InterquartileRange |
|  | gradient_glszm_LargeAreaLowGrayLevelEmphasis |
|  | logarithm_gldm_DependenceEntropy |
|  | logarithm_firstorder_RobustMeanAbsoluteDeviation |
|  | logarithm_gldm_HighGrayLevelEmphasis |
|  | logarithm_glszm_SmallAreaHighGrayLevelEmphasis |
|  | wavelet-H_glrlm_RunLengthNonUniformity |
|  | log-sigma-1-0-mm-3D_glrlm_RunLengthNonUniformity |
|  | wavelet-H_glszm_LargeAreaHighGrayLevelEmphasis |
|  | gradient_gldm_DependenceEntropy |
|  | log-sigma-1-0-mm-3D_gldm_DependenceEntropy |
|  | wavelet-H_glcm_JointAverage |
|  | wavelet-L_firstorder_Minimum |
|  | logarithm_glrlm_HighGrayLevelRunEmphasis |
|  | logarithm_firstorder_90Percentile |
|  | square_glcm_JointEntropy |
|  | gradient_glrlm_ShortRunHighGrayLevelEmphasis |
|  | squareroot_ngtdm_Strength |
|  | logarithm_glrlm_ShortRunHighGrayLevelEmphasis |
| 2D DL (N =15) | DL_256 |
|  | DL_514 |
|  | DL_491 |
|  | DL_6 |
|  | DL_732 |
|  | DL_418 |
|  | DL_96 |
|  | DL_472 |
|  | DL_702 |
|  | DL_300 |
|  | DL_523 |
|  | DL_296 |
|  | DL_27 |
|  | DL_245 |
|  | DL_527 |
